# Supplementary material for: Spatiotemporal dynamics of macrophage heterogeneity and a potential function of Trem2hi macrophages in infarcted hearts
Source: Nat Commun. 2022 Aug 6;13:4580. doi: 10.1038/s41467-022-32284-2 (PMC9357004; doi:10.1038/s41467-022-32284-2)
Supplement: Supplementary file 1 — Supplementary Information [file 41467_2022_32284_MOESM1_ESM.pdf]

Supplementary Information

Spatiotemporal dynamics of macrophage heterogeneity and a potential function of Trem2^{hi} macrophages in infarcted hearts

Seung-Hyun Jung[1,2,9*], Byung-Hee Hwang[3,4,9], Sun Shin[5,9], Eun-Hye Park[3], Sin-Hee Park[3], Chan Woo Kim[3], Eunmin Kim[3], Eunho Choo[3,4], Ik Jun Choi[6], Filip K Swirski[7], Kiyuk Chang[3,4*], Yeun-Jun Chung[2,5,8*]

[1]Department of Biochemistry, College of Medicine, The Catholic University of Korea; [2]Department of Biomedicine & Health Sciences, College of Medicine, The Catholic University of Korea; [3]Cardiovascular Research Institute for Intractable Disease, College of Medicine, The Catholic University of Korea; [4]Division of Cardiology, Seoul St. Mary's Hospital, College of Medicine, The Catholic University of Korea; [5]Departments of Microbiology, IRCGP, Cancer Evolution Research Center, College of Medicine, The Catholic University of Korea; [6]Division of Cardiology, Incheon St. Mary's Hospital, College of Medicine, The Catholic University of Korea; [7]Cardiovascilar Research Institute, Icahn School of Medicine at Mount Sinai, New York, NY, USA; [8]Precision Medicine Research Center, The Catholic University of Korea; [9]These authors contributed equally to this work.

Contents:

Supplementary figures 1-16

Supplementary Tables 1-3

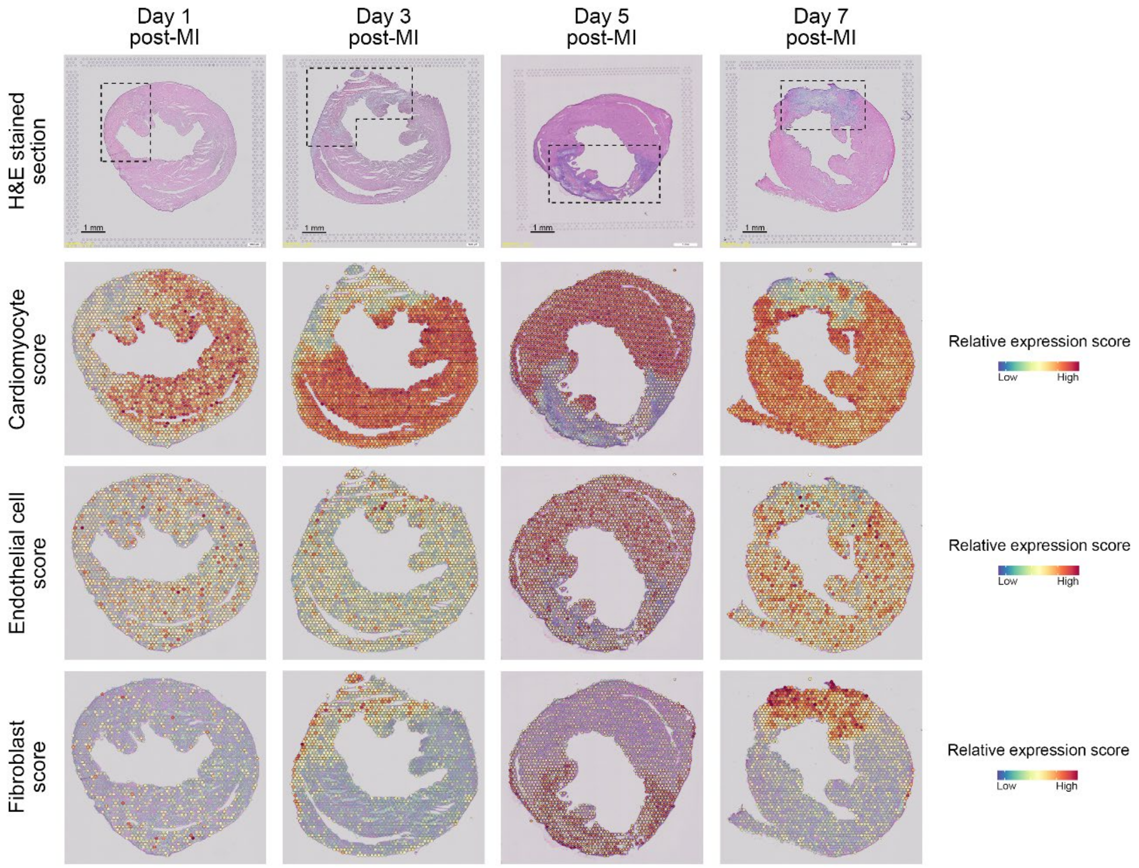

Supplementary Figure 1. Spatial transcriptome sequencing (ST-seq) of mouse heart after MI. The first row represents H&E-stained images containing the infarcted area (dotted box). The last three rows represent cell-type-specific scores calculated by taking the mean of the scaled and centered expression value across multiple signature genes. Results are representative of four different samples. Blue and red indicate lower and higher expression, respectively.

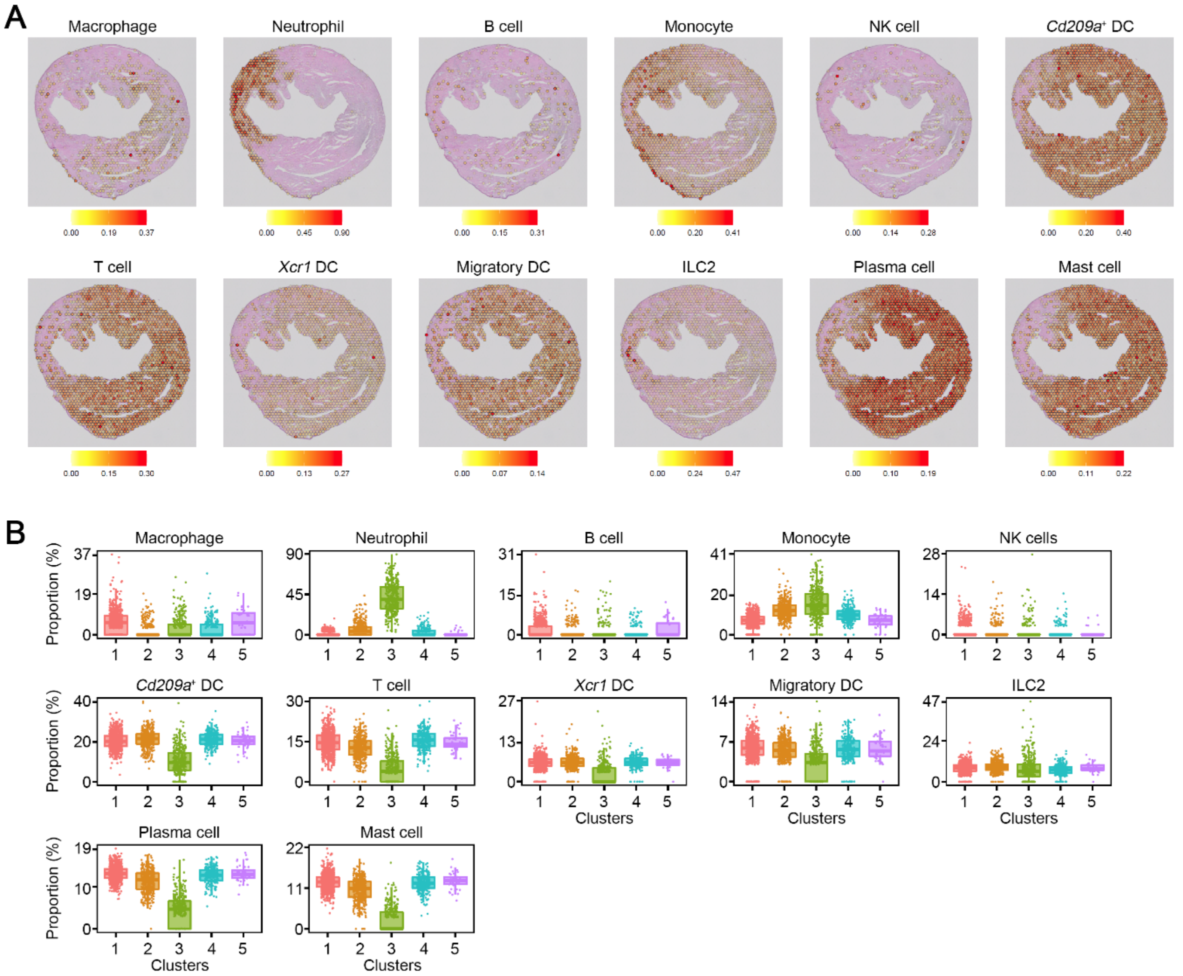

Supplementary Figure 2. Spatial heterogeneity of infiltrated immune cells into mouse heart. (A) Sample day 1 post-MI predicted cell type proportion within each spot showing spatially differential immune patterns. The scale bar is marked on the H&E stained section in Supplementary Figure 1. (B) Box plots of the cell types for day 1 post-MI. Box plots displaying differences in cell type proportion of clusters which corresponds main figure 2b. Each cluster (cluster 1 – 5) has the same number of spots (n=1,803 spots). The lower whisker, lower hinge, box center, upper hinge, and upper whisker represent the minimum, lower quartile, median, upper quartile, and maximum calculated without outlier values which are more than 1.5x interquartile range of the lower and upper quartiles. Source data are provided in the Source Data file.

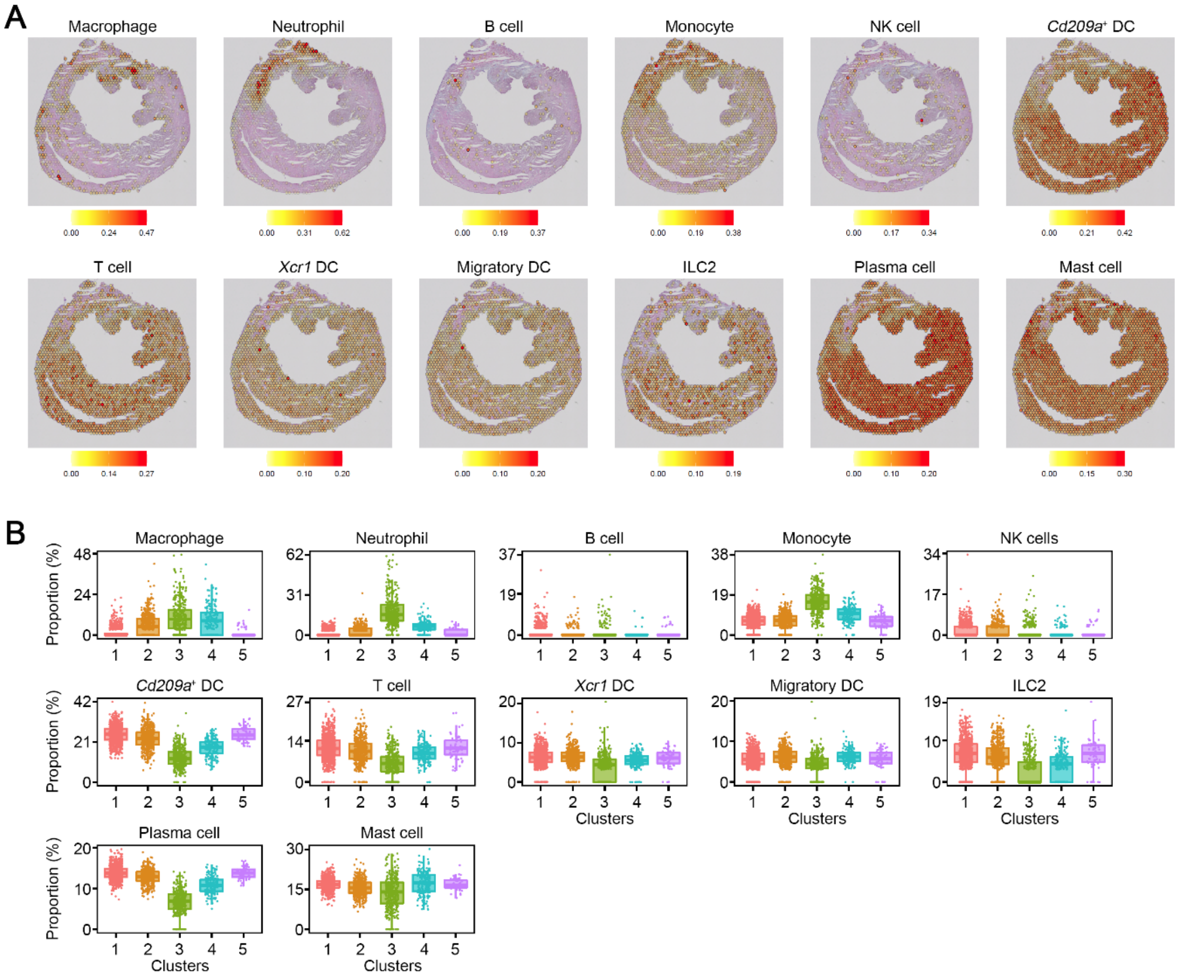

Supplementary Figure 3. Spatial heterogeneity of infiltrated immune cells into mouse heart. (A) Sample day 3 post-MI predicted cell type proportion within each spot showing spatially differential immune patterns. The scale bar is marked on the H&E stained section in Supplementary Figure 1. (B) Box plots of the cell types for day 3 post-MI. Box plots displaying differences in cell type proportion of clusters which corresponds main figure 2b. Each cluster (cluster 1 – 5) has the same number of spots (n=1,916 spots). The lower whisker, lower hinge, box center, upper hinge, and upper whisker represent the minimum, lower quartile, median, upper quartile, and maximum calculated without outlier values which are more than 1.5x interquartile range of the lower and upper quartiles. Source data are provided in the Source Data file.

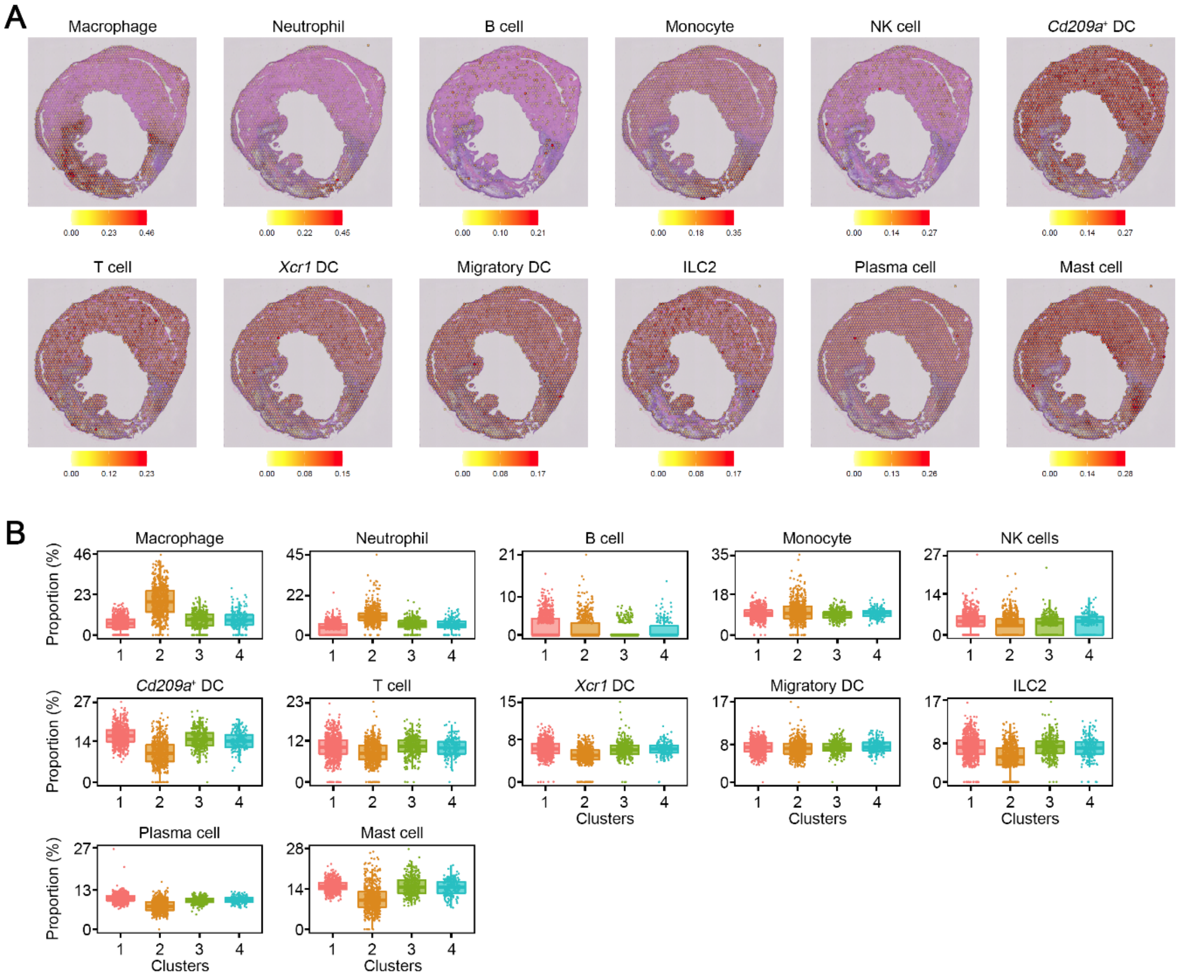

Supplementary Figure 4. Spatial heterogeneity of infiltrated immune cells into mouse heart. (A) Sample day 5 post-MI predicted cell type proportion within each spot showing spatially differential immune patterns. The scale bar is marked on the H&E stained section in Supplementary Figure 1. (B) Box plots of the cell types for day 5 post-MI. Box plots displaying differences in cell type proportion of clusters which corresponds main figure 2b. Each cluster (cluster 1 – 4) has the same number of spots (n=1,870 spots). The lower whisker, lower hinge, box center, upper hinge, and upper whisker represent the minimum, lower quartile, median, upper quartile, and maximum calculated without outlier values which are more than 1.5x interquartile range of the lower and upper quartiles. Source data are provided in the Source Data file.

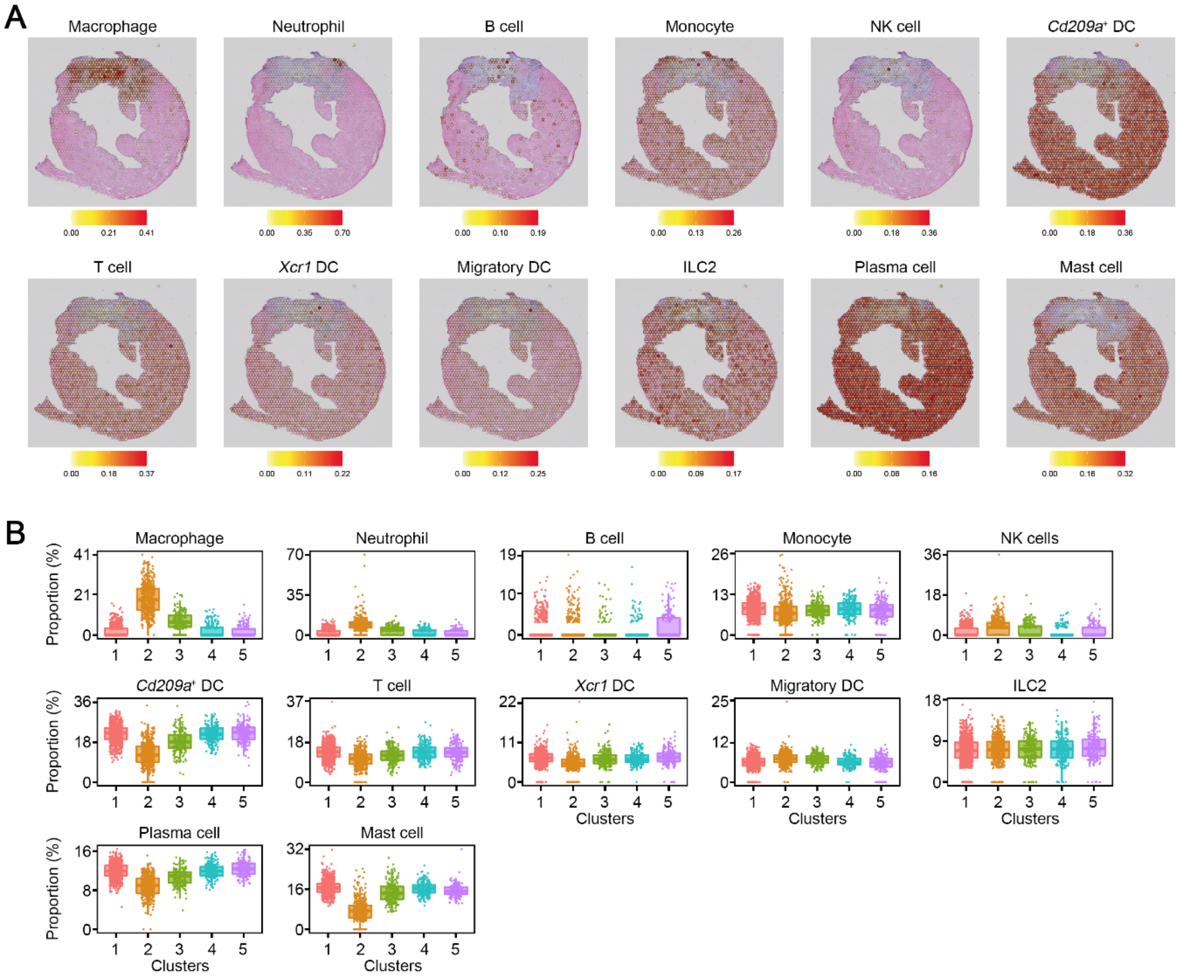

Supplementary Figure 5. Spatial heterogeneity of infiltrated immune cells into mouse heart. (A) Sample day 7 post-MI predicted cell type proportion within each spot showing spatially differential immune patterns. The scale bar is marked on the H&E stained section in Supplementary Figure 1. (B) Box plots of the cell types for day 7 post-MI. Box plots displaying differences in cell type proportion of clusters which corresponds main figure 2b. Each cluster (cluster 1 – 5) has the same number of spots (n=1,973 spots). The lower whisker, lower hinge, box center, upper hinge, and upper whisker represent the minimum, lower quartile, median, upper quartile, and maximum calculated without outlier values which are more than 1.5x interquartile range of the lower and upper quartiles. Source data are provided in the Source Data file.

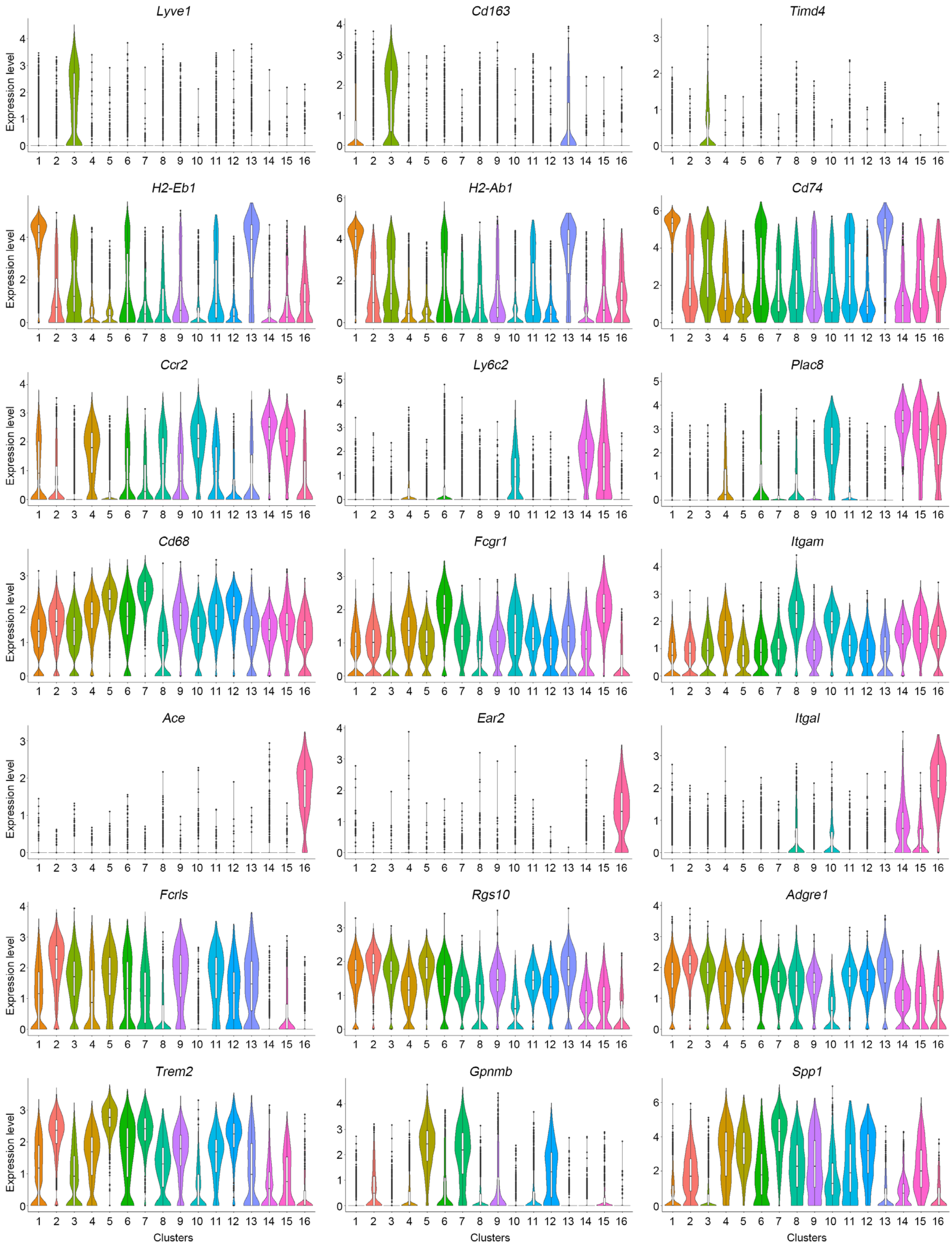

Supplementary Figure 6. Violin plots of typical marker genes strongly and specifically associated with each macrophage sub-cluster (Cluster 1: 3,158 cells; Cluster 2: 2,591 cells; Cluster 3: 2,498 cells; Cluster 4: 1,830 cells; Cluster 5: 1,789 cells; Cluster 6: 1,389 cells;

Cluster 7: 1,223 cells; Cluster 8: 1,168 cells; Cluster 9: 1,146 cells; Cluster 10: 1,008 cells; Cluster 11: 860 cells; Cluster 12: 755 cells; Cluster 13: 691 cells; Cluster 14: 632 cells; Cluster 15: 467 cells; Cluster 16: 328 cells). The lower whisker, lower hinge, box center, upper hinge, and upper whisker represent the minimum, lower quartile, median, upper quartile, and maximum calculated without outlier values which are more than 1.5x interquartile range of the lower and upper quartiles. Source data are provided in the Source Data file.

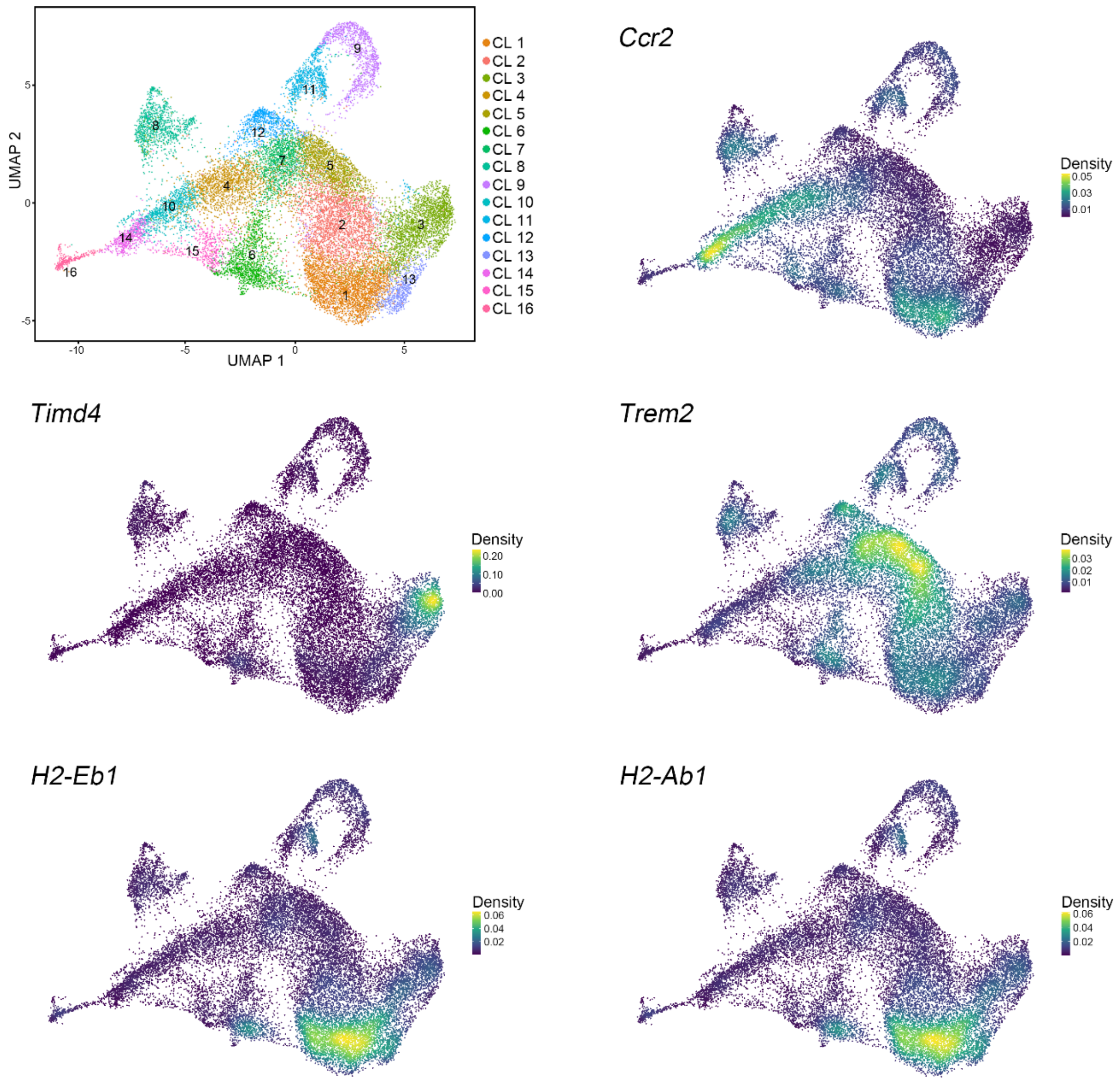

Supplementary Figure 7. Distribution of marker genes for monocyte/macrophage sub-clusters using the Nebulosa package (https://github.com/powellgenomicslab/Nebulosa).

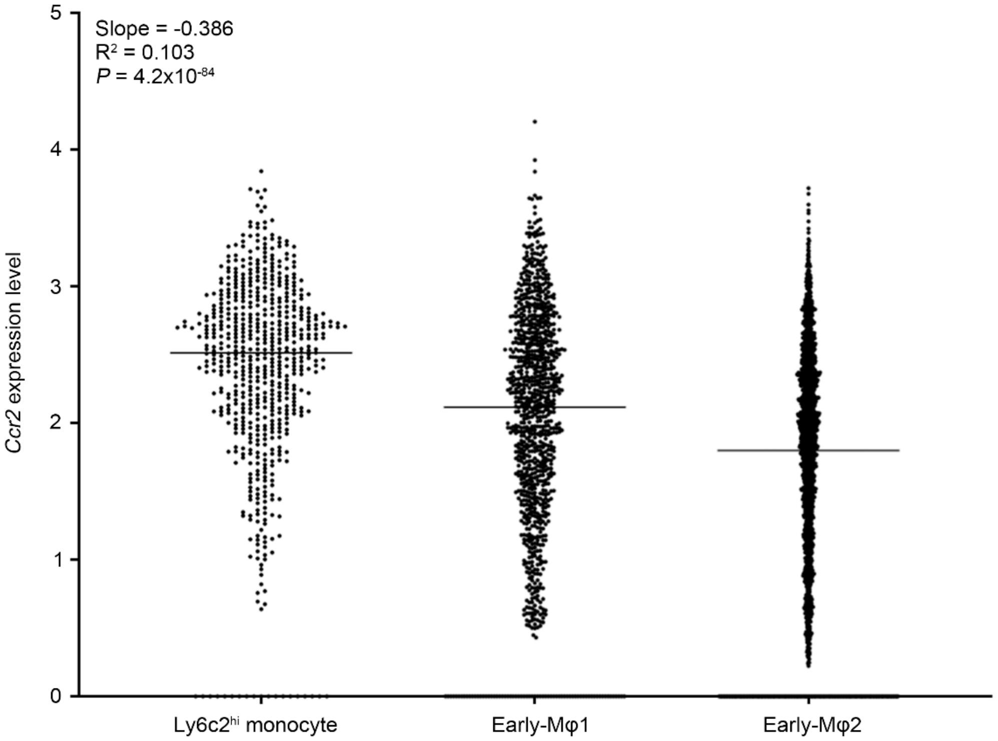

Supplementary Figure 8. Ccr2 expression level across Ly6c2hi monocyte (n=632 cells), Early-Mφ1 (n=1,008 cells), and Early-Mφ2 (n=1,830 cells). The expression of *Ccr2* was inversely correlated between Ly6c2hi monocytes, and Early-Mφ1 and Early-Mφ2 cells (R^2 = 0.103, $P = 4.2x10^{-84}$, linear regression analysis). Each dot represents single cell. Source data are provided in the Source Data file.

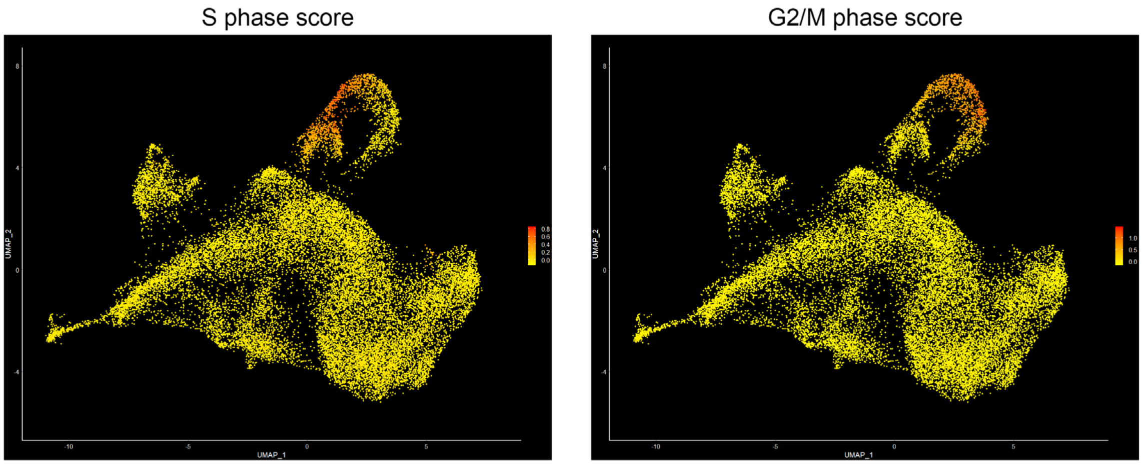

Supplementary Figure 9. Expression levels of S phase and G2/M are shown. Cell cycle analysis was performed by using the "CellCycleScoring" function in Seurat. Yellow and red indicate lower and higher expression, respectively.

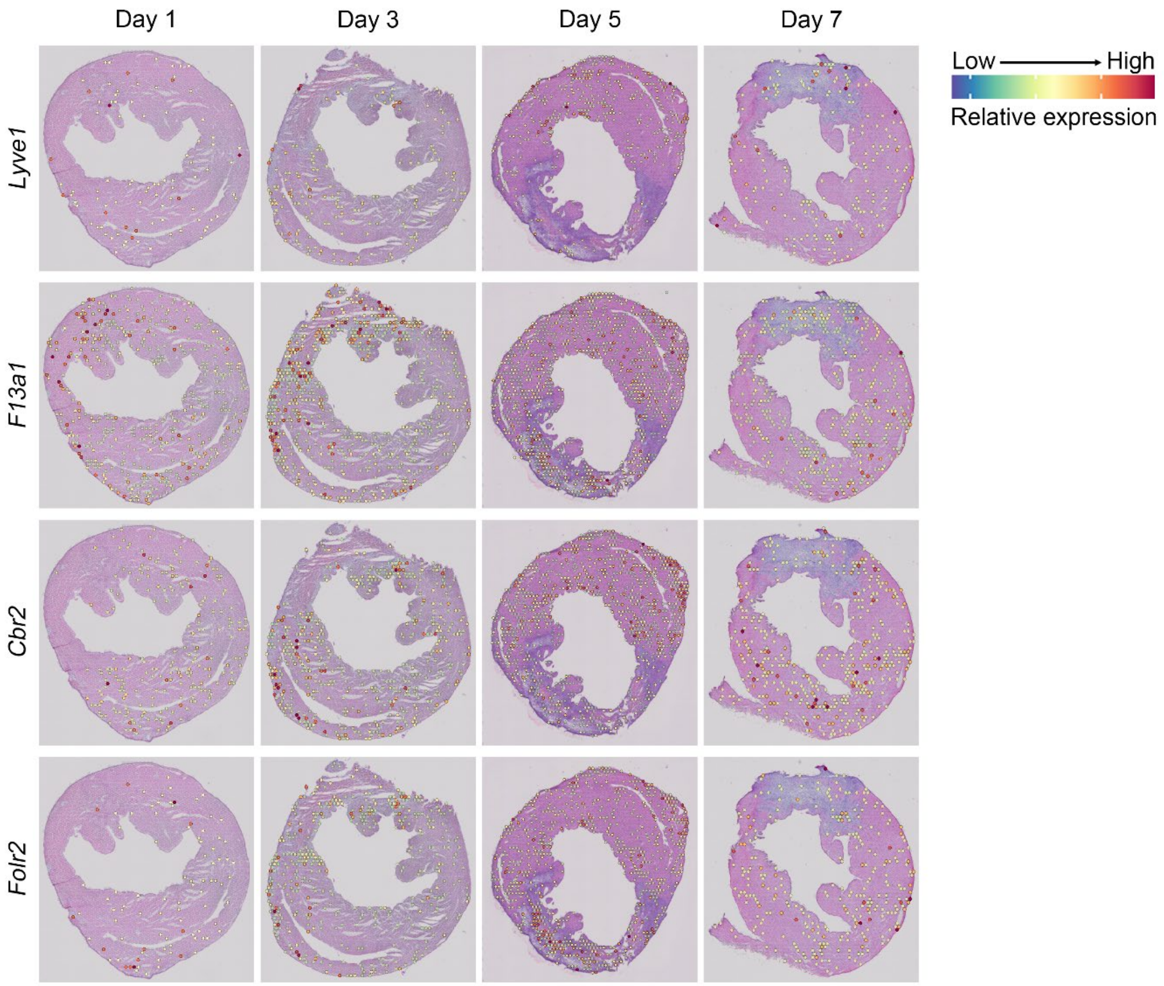

Supplementary Figure 10. Expression levels of marker genes (*Lyve1*, *F13a1*, *Cbr2*, *Folr2*) for SS-Mφ1 by ST-seq. Blue and red indicate lower and higher expression, respectively. SS-Mφ1 cells were dispersed in the heart with respect to all time points after MI. The scale bar is marked on the H&E stained section in Supplementary Figure 1. Results are representative of four different samples.

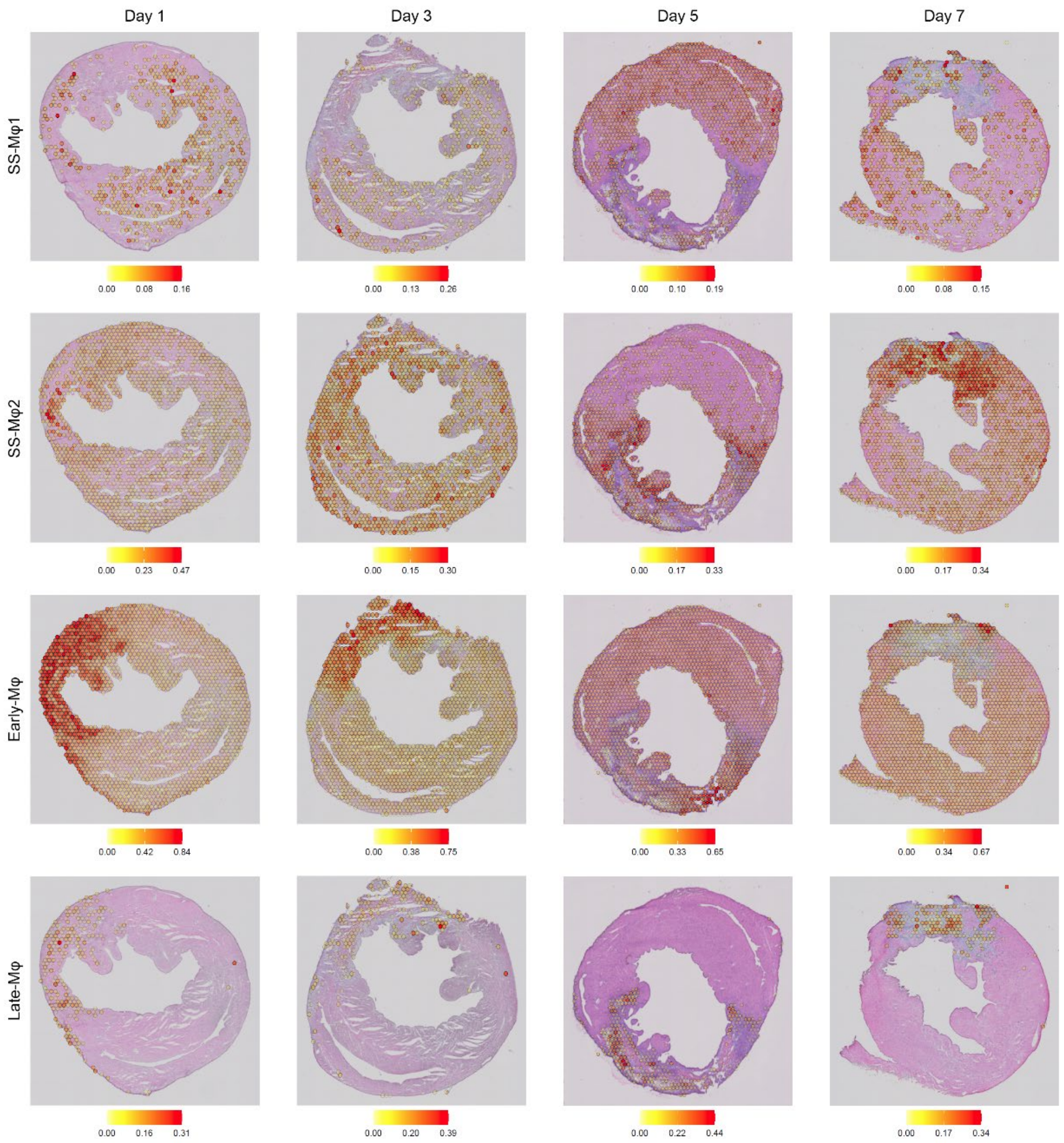

Supplementary Figure 11. Spatial heterogeneity of infiltrated monocyte/macrophage subsets into mouse heart. Marker genes of monocyte/macrophage sub-clusters from scRNA-seq were used to train the SPOTlight analysis. Each dot color (yellow to red) represents the proportion of each monocyte/macrophage subsets. The scale bar is marked on the H&E stained section in Supplementary Figure 1. Results are representative of four different samples.

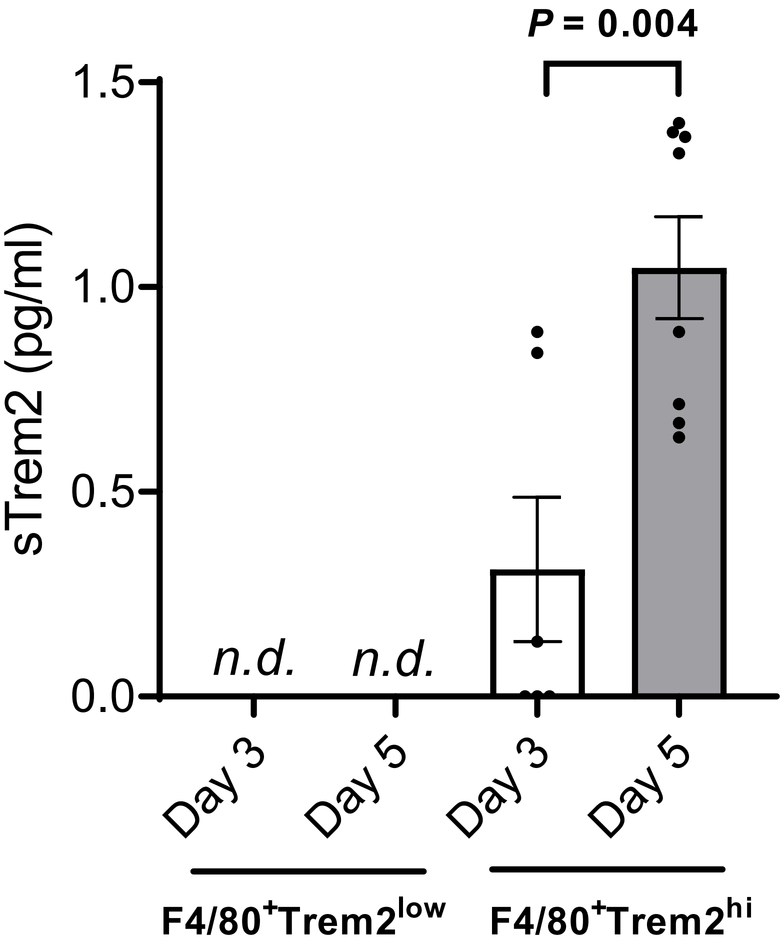

Supplementary Figure 12. Secretion levels of sTrem2 in the late stage Trem2^{hi} macrophages. F4/80^{+}Trem2^{hi} and F4/80^{+}Trem2^{low} macrophage subsets were sorted from the infarcted heart tissue on days 3 (F4/80^{+}Trem2^{low}, n=8; F4/80^{+}Trem2^{hi}, n=6) and 5 post-MI (F4/80^{+}Trem2^{low}, n=8; F4/80^{+}Trem2^{hi}, n=8). The sorted cells were cultured for 2 hours and the secretion levels of sTrem2 was measured by ELISA. Unpaired two-tailed t-test was used to determine the statistical significance. n.d.: not detected. Data are presented as mean ± SEM. Source data are provided in the Source Data file.

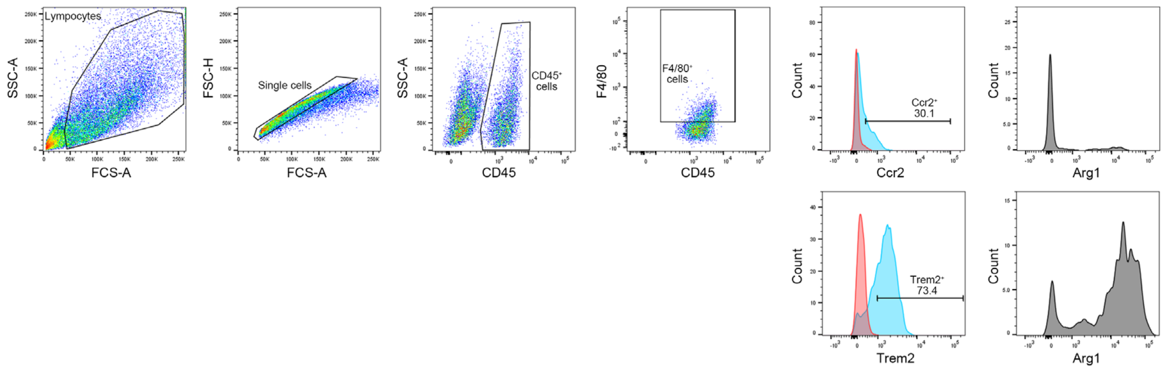

Supplementary Figure 13. Single cell suspensions obtained from the heart tissue were analyzed by flow cytometry. The gates and regions are placed around populations of cells with common characteristics, usually forward scatter (FSC), side scatter (SSC). To collect Trem2^{hi} macrophage subsets, we sorted the immune cells based on CD45 expression, and the macrophage population was selected using F4/80. Ccr2^{hi} macrophage and Trem2^{hi} macrophage, as major macrophage subset in the early and late phases, were then collected from the CD45^{+}F4/80^{+} macrophages, respectively. To determine the polarizing status of Trem2^{hi} macrophage subsets, we analyzed the expression level of M2 macrophage marker Arginase1 (Arg1).

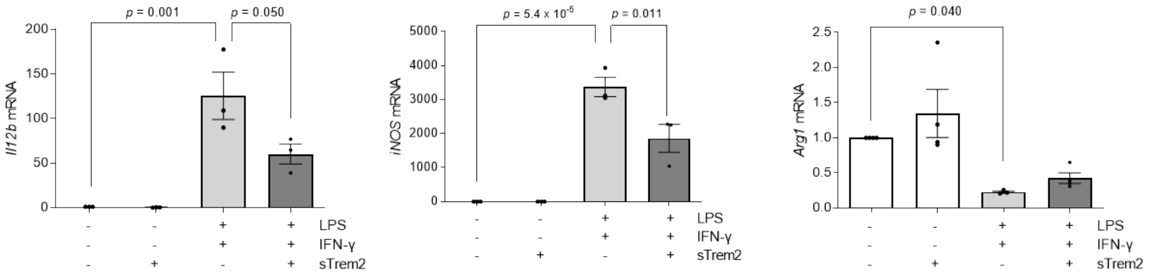

Supplementary Figure 14. sTrem2-induced effects on macrophage polarization *in vitro*. Thioglycolate-elicited peritoneal macrophages from C57BL/6 mice were sorted and polarized to a pro-inflammatory phenotype by lipopolysaccharide (LPS, 1 μg/mL) and interferon-γ (IFN-γ, 4 ng/mL) treatments. The expression levels of *Il12b* (n=3) and *Nos2* (pro-inflammatory markers; n=3) were upregulated while *Arg1* (anti-inflammatory marker; n=4) was downregulated in a pro-inflammatory phenotype. On the contrary, sTrem2 (200 ng/mL) treated pro-inflammatory phenotype macrophages showed decreased expression levels of *Il12b* (n=3) and *Nos2* (n=3) and increased expression levels of *Arg1* (n=4). One-way ANOVA with Turkey's test was used to determine the statistical significance. Data are presented as mean ± SEM. Source data are provided in the Source Data file.

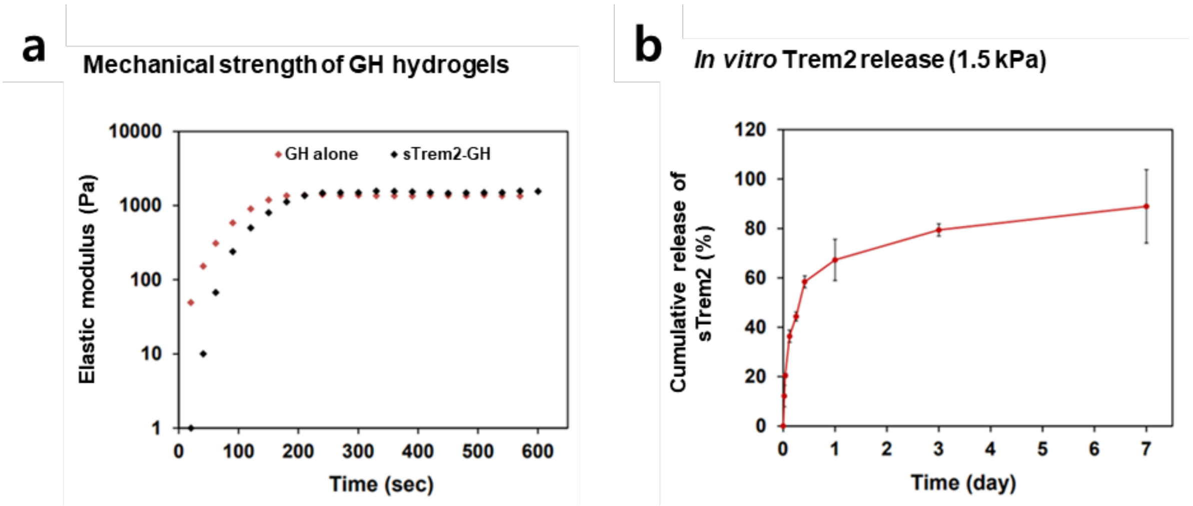

Supplementary Figure 15. Mechanical strength of Gelatin-Hydroxyphenyl propionic acid (GH) hydrogels and in vitro Trem2 release profile at the condition of 1.5 kPa elastic modulus. (a) Mechanical strength of GH hydrogels. By adjusting the cross-linking densities of GH hydrogels, the elastic modulus of GH only and sTrem2-GH was fixed at 1.5 kPa. (b) In vitro Trem2 release profile at the condition of 1.5 kPa elastic modulus. In vitro release test of sTrem2-GH revealed that 70% of sTrem2 protein was released out from sTrem2-GH by day 1 and approximately 90% of cumulative release in a sustained manner was evaluated by 7 days (n=3). Data are presented as mean ± SD. Source data are provided in the Source Data file.

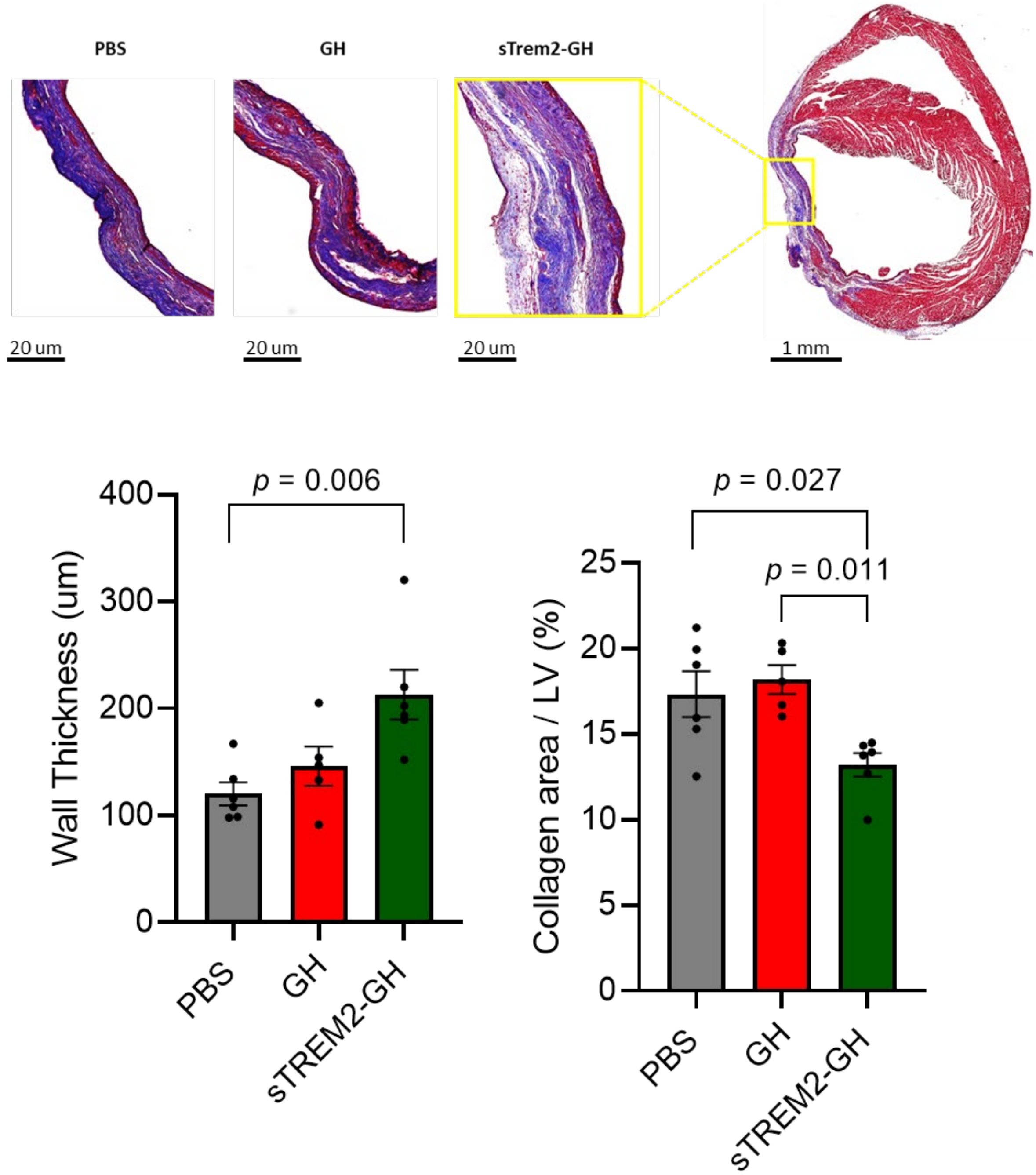

Supplementary Figure 16. Masson's trichrome (MT) stained images of heart tissues of PBS-, GH-, and sTrem2-GH-treated groups on day 28 post-MI (results are representative of three different samples) and quantitative analysis of wall thickness and collagen area in the infarcted tissue (PBS-, n=6; GH-, n=5; sTrem2-GH, n=6). One-way ANOVA with Turkey's test was used to determine the statistical significance. Data are validated using independent samples and presented as mean ± SEM. Source data are provided in the Source Data file.

Supplementary Table 1. The portions of cells in each of the 12 broad cell types according to the time-point after MI.

	Infarct size (%)*	Macrophage	Neutrophil	B cell	Monocyte	NK cell	Cd209a+ DC	T cell	Xcr1 DC	Migratory DC	ILC2	Plasma cell	Mast cell	Total
Steady state	-	3,860	204	1,196	245	568	119	281	37	4	57	3	7	6,581
		58.7%	3.1%	18.2%	3.7%	8.6%	1.8%	4.3%	0.6%	0.1%	0.9%	0.0%	0.1%	100%
Day 1 post-MI	29.91 ± 5.84	1,831	4,009	384	564	87	250	164	48	11	14	0	1	7,363
		24.9%	54.4%	5.2%	7.7%	1.2%	3.4%	2.2%	0.7%	0.1%	0.2%	0.0%	0.0%	100%
Day 3 post-MI	31.65 ± 4.64	2,968	660	99	284	65	272	18	35	42	2	0	1	4,446
		66.8%	14.8%	2.2%	6.4%	1.5%	6.1%	0.4%	0.8%	0.9%	0.0%	0.0%	0.0%	100%
Day 5 post-MI	33.18 ± 1.52	6,110	126	75	259	84	440	23	61	67	4	19	3	7,271
		84.0%	1.7%	1.0%	3.6%	1.2%	6.1%	0.3%	0.8%	0.9%	0.1%	0.3%	0.0%	100%
Day 7 post-MI	33.58 ± 3.08	7,032	136	81	155	177	384	107	87	64	28	47	18	8,316
		84.6%	1.6%	1.0%	1.9%	2.1%	4.6%	1.3%	1.0%	0.8%	0.3%	0.6%	0.2%	100%

*Infarct sizes are shown as the mean ± SD (n = 4).

Supplementary Table 2. Echocardiographic parameters.

	PBS (n=6)	GH (n=7)	sTrem2-GH (n=7)
**IVSd**	0.28 ± 0.66	0.29 ± 0.04 **	0.36 ± 0.07***
**LVIDd**	5.97 ± 0.53	5.62 ± 0.67	5.31 ± 0.20***
**LVPWd**	0.53 ± 0.09	0.57 ± 0.03	0.54 ± 0.04
**IVSs**	0.31 ± 0.01	0.34 ± 0.07	0.43 ± 0.08
**LVIDs**	5.55 ± 0.58	5.07 ± 0.78	4.21 ± 0.33
**LVPWs**	0.63 ± 0.02	0.65 ± 0.06	0.65 ± 0.05

Interventricular septal thickness at diastole (IVSd), left ventricle internal dimension at diastole (LVIDd), left ventricle posterior wall thickness at diastole (LVPWd), interventricular septal thickness at systole (IVSs), left ventricle internal dimension at systole (LVIDs), and left ventricle posterior wall thickness at systole (LVPWs). Data are shown as the mean ± SD. **P<0.01 and ***P<0.001 indicate significant differences compared to PBS-treated mice at day 28 post-MI. One-way ANOVA with Turkey's test was used to determine the statistical significance. Source data with the exact P values are provided in the Source Data file.

Supplementary Table 3. Primer list used in qRT-PCR analyses.

Gene	Accession number	Forward (5' to 3')	Reverse (5' to 3')	size (bp)
Il1b	NM_008361.4	CTTTCCCGTGGACCTTCCAG	ATGGGAACGTCACACACCAG	124
Il6	NM_031168	CACTTCACAAGTCGGAGGCT	CTGCAAGTGCATCATCGTTGT	113
Il10	NM_010548.2	TGAGGCGCTGTCATCGATTT	TGGCCTTGTAGACACCTTGG	105
Alox15	NM_009660.3	TCAAGAGTGGCCACACCAAG	GTAGACCGCTTCAGCACCAT	115
Cx3cr1	NM_009987	GTCTTCACGTTCGGTCTGGT	ACAAAGAGCAGGTCGCTCAA	122
Cxcr3	NM_009910	AGCTAGTGGCTGGTTTCCTG	GCCTCATAGCTCGAAAACGC	111
Cxcr7	NM_001271607	ACTCGGCCAAAACAGGTCTC	CGACCTCTGCAGAATGATGGA	110
Ccl2	NM_011333.3	CAAGAAGGAATGGGTCCAGA	GCTGAAGACCTTAGGGCAGA	105
Tgfb1	NM_011577	CTGCTGACCCCCACTGATAC	GTGAGCGCTGAATCGAAAGC	111
Spp1	NM_001204203	CCGAGGTGATAGCTTGGCTT	GGACTCCTTAGACTCACCGC	130
