# Supplementary material for: Spatiotemporal dynamics of macrophage heterogeneity and a potential function of Trem2hi macrophages in infarcted hearts
Source: Nat Commun. 2022 Aug 6;13:4580. doi: 10.1038/s41467-022-32284-2 (PMC9357004; doi:10.1038/s41467-022-32284-2)
Supplement: Supplementary file 3 — Description of Additional Supplementary Files [file 41467_2022_32284_MOESM3_ESM.docx]

**Description of Additional Supplementary Files**

File Name: Supplementary Data 1.

Description: Significantly enriched genes in each 12 broad cell cluster. Logistic regression framework was used to determine differentially expressed genes. “Avg_logFC” is log fold-change of the average expression between the two groups. Positive values indicate that the feature is more highly expressed in the target cluster. “pct.1” is the percentage of cells where the feature is detected in the target cluster. “pct.2” is the percentage of cells where the feature is detected in the other clusters. “p_val_adj” is an adjusted p-value, based on bonferroni correction using all features in the dataset.

File Name: Supplementary Data 2.

Description: Significantly enriched genes in each macrophage subset. Logistic regression framework was used to determine differentially expressed genes. “Avg_logFC” is log fold-change of the average expression between the two groups. Positive values indicate that the feature is more highly expressed in the target cluster. “pct.1” is the percentage of cells where the feature is detected in the target cluster. “pct.2” is the percentage of cells where the feature is detected in the other clusters. “p_val_adj” is an adjusted p-value, based on bonferroni correction using all features in the dataset.

File Name: Supplementary Data 3.

Description: Significantly enriched genes in each neutrophil subset. Logistic regression framework was used to determine differentially expressed genes. “Avg_logFC” is log fold-change of the average expression between the two groups. Positive values indicate that the feature is more highly expressed in the target cluster. “pct.1” is the percentage of cells where the feature is detected in the target cluster. “pct.2” is the percentage of cells where the feature is detected in the other clusters. “p_val_adj” is an adjusted p-value, based on bonferroni correction using all features in the dataset.

File Name: Supplementary Data 4.

Description: Significantly enriched genes in each dendritic cell subset. Logistic regression framework was used to determine differentially expressed genes. “Avg_logFC” is log fold-change of the average expression between the two groups. Positive values indicate that the feature is more highly expressed in the target cluster. “pct.1” is the percentage of cells where the feature is detected in the target cluster. “pct.2” is the percentage of cells where the feature is detected in the other clusters. “p_val_adj” is an adjusted p-value, based on bonferroni correction using all features in the dataset.

File Name: Supplementary Movie 1:

Description: Echocardiogram of mouse treated with PBS. Parasternal short axis view at the papillary muscle level was obtained to evaluate the LV wall motion at day 28 post-MI.

File Name: Supplementary Movie 2: Echocardiogram of mouse treated with GH alone. Parasternal short axis view at the papillary muscle level was obtained to evaluate the LV wall motion at day 28 post-MI.

File Name: Supplementary Movie 3:

Description: Echocardiogram of mouse treated with sTrem2-GH. Parasternal short axis view at the papillary muscle level was obtained to evaluate the LV wall motion at day 28 post-MI.
