# Supplementary material for: Spatiotemporal dynamics of macrophage heterogeneity and a potential function of Trem2hi macrophages in infarcted hearts
Source: Nat Commun. 2022 Aug 6;13:4580. doi: 10.1038/s41467-022-32284-2 (PMC9357004; doi:10.1038/s41467-022-32284-2)
Supplement: Supplementary file 11 — Reporting Summary [file 41467_2022_32284_MOESM11_ESM.pdf]

nature portfolio

Corresponding author(s): Yeun-Jun Chung
Last updated by author(s): Jun 28, 2022

Reporting Summary

Nature Portfolio wishes to improve the reproducibility of the work that we publish. This form provides structure for consistency and transparency in reporting. For further information on Nature Portfolio policies, see our Editorial Policies and the Editorial Policy Checklist.

Statistics

For all statistical analyses, confirm that the following items are present in the figure legend, table legend, main text, or Methods section.

n/a	Confirmed	
☐	☒	The exact sample size (*n*) for each experimental group/condition, given as a discrete number and unit of measurement
☐	☒	A statement on whether measurements were taken from distinct samples or whether the same sample was measured repeatedly
☐	☒	The statistical test(s) used AND whether they are one- or two-sided<br>*Only common tests should be described solely by name; describe more complex techniques in the Methods section.*
☒	☐	A description of all covariates tested
☒	☐	A description of any assumptions or corrections, such as tests of normality and adjustment for multiple comparisons
☐	☒	A full description of the statistical parameters including central tendency (e.g. means) or other basic estimates (e.g. regression coefficient) AND variation (e.g. standard deviation) or associated estimates of uncertainty (e.g. confidence intervals)
☐	☒	For null hypothesis testing, the test statistic (e.g. *F*, *t*, *r*) with confidence intervals, effect sizes, degrees of freedom and *P* value noted<br>*Give P values as exact values whenever suitable.*
☒	☐	For Bayesian analysis, information on the choice of priors and Markov chain Monte Carlo settings
☒	☐	For hierarchical and complex designs, identification of the appropriate level for tests and full reporting of outcomes
☒	☐	Estimates of effect sizes (e.g. Cohen's *d*, Pearson's *r*), indicating how they were calculated

Our web collection on statistics for biologists contains articles on many of the points above.

Software and code

Policy information about availability of computer code

Data collection	Echocardiography: Affinity 50 imaging system (Philips); FACS-sorting: FACSCanto II Flow Cytometry System (BD); Single-cell capture: 10x Genomic Chromium System (10x Genomic); single-cell RNA sequencing: HiSeq2500 platform (Illumina); Spatial transcriptome: Visium Spatial Gene Expression system (10X Genomics); Fluorescent imaging: LSM700 and LSM800 (Carl Zeiss); Brightfield imaging: BX51 (Olympus); spatial transcriptome sequencing: NovaSeq 6000 system (Illumina); Infarct size determination: digital pathology scanner (Aperio AT Turbo); Western blotting: chemiluminescence system (Amersham); qRT-PCR: CFX96 Real-Time PCR detection system (BIO-RAD Laboratories)
Data analysis	Flow-jo software v10, Cell Ranger v3.0.1, Seurat v3.2.0, SingleR v1.2.4, Monocle3 v.0.2.3, Space Ranger v1.0.0, SPOTlight v0.1.7, Nubulosa v1.7.0, OlyVia software v2.2, ImageJ software v1.8.0, Bio-Rad CFX Manager v3.1. Relevant codes used for data analysis are available from https://github.com/Junglab-CMC/Macrophage-heterogeneity-after-MI.

For manuscripts utilizing custom algorithms or software that are central to the research but not yet described in published literature, software must be made available to editors and reviewers. We strongly encourage code deposition in a community repository (e.g. GitHub). See the Nature Portfolio guidelines for submitting code & software for further information.

Data

Policy information about availability of data

All manuscripts must include a data availability statement. This statement should provide the following information, where applicable:

- Accession codes, unique identifiers, or web links for publicly available datasets
- A description of any restrictions on data availability
- For clinical datasets or third party data, please ensure that the statement adheres to our policy

The scRNA-seq and ST-seq data sets generated in this study were deposited in Gene Expression Omnibus under the accession numbers GSE163129 and GSE165857,

respectively. Sequencing reads were mapped to the mm10 version 3.0.0 reference, downloaded from the 10x Genomics (https://support.10xgenomics.com/single-cell-gene-expression/software/downloads/latest).

Field-specific reporting

Please select the one below that is the best fit for your research. If you are not sure, read the appropriate sections before making your selection.

- [x] Life sciences
- [ ] Behavioural & social sciences
- [ ] Ecological, evolutionary & environmental sciences

For a reference copy of the document with all sections, see nature.com/documents/nr-reporting-summary-flat.pdf

Life sciences study design

All studies must disclose on these points even when the disclosure is negative.

Sample size	No sample size calculation was performed for this study. To verify the in vivo effects, 5-10 animals in each group are generally used for the comparison in major journals because differences between group are usually marked in animal experiment, not like human experiments (Arifin WN et al. 2017, Malays J Med Sci; Charan J et al. 2013, J Pharmacol Pharmacother). To verify the in vivo effect of sTrem2 on the remodeling after MI, we injected sTrem2 mixed with an injectable gelatin hydrogel (GH). The primary objective of this experiment was to compare the functional and structural improvements of infarcted hearts. Comparative analysis in cardiac function was performed with the following groups: Sham operation (n=7), PBS-treated group (n=6), GH-treated group (n=7), and sTrem2-GH-treated goup (n=7). For comparative analysis in cardiac structure (infarct size), we compared the following three groups: PBS-treated group (n=8), GH-treated group (n=7), and sTrem2-GH-treated goup (n=8). For survival analysis, four groups were compared and each group was comprised of 5-12 mice: sham operation group (n=5), PBS-treated group (n=11), GH-treated group (n=11), and sTrem2-GH-treated goup (n=12).
Data exclusions	We did not exclude any samples from immunohistochemistry, western blot, and Flow cytometric analysis.
Replication	The results of IHC, WB, flow cytometry, echocardiography, and survival analysis were obtained by measuring biologically independent samples and exact numbers were provided in the manuscript. Gene expression analysis by qRT-PCR was repeated five (F4/80+Ccr2+ macrophages at day 1 post-MI) or six (F4/80+Trem2+ macrophages at day 5 post-MI) times to verify the reproducibility. All obtained results were uploaded the "Source Data.xlsx"
Randomization	We randomly allocated MI mice into three difference groups.
Blinding	Three different and blinded investigators were participated in 1) group allocation and treatment, 2) histology and molecular work, and 3) data analysis during experiment. For echocardiographic analysis, the investigators ensured unpredictability of allocation by a random process, in which the investigators were unaware of which treatment mice was received.

Reporting for specific materials, systems and methods

We require information from authors about some types of materials, experimental systems and methods used in many studies. Here, indicate whether each material, system or method listed is relevant to your study. If you are not sure if a list item applies to your research, read the appropriate section before selecting a response.

Materials & experimental systems

n/a	Involved in the study	
	x	Antibodies
x		Eukaryotic cell lines
x		Palaeontology and archaeology
	x	Animals and other organisms
x		Human research participants
x		Clinical data
x		Dual use research of concern

Methods

n/a	Involved in the study	
x		ChIP-seq
	x	Flow cytometry
x		MRI-based neuroimaging

Antibodies

Antibodies used	We purchased FITC anti mouse CD45 antibody (BD, CatNo: 553080, LotNo: 51533) and BV421 anti mouse Ccr2 (BioLegend, CatNo: 150605, LotNo: B284305), APC anti human/mouse Trem2 (R&D system, CatNo: FAB17291A, LotNo: AADS0619041), PE-Cy7 anti human/mouse Arg-1 (Invitrogen, CatNo: 25-3697-82, LotNo: 2053279), anti-TREM2 (abcam, CatNo: ab175525, LotNo: GR3261360-3), anti-CD68 (Abcam, CatNo: ab53444, LotNo: GR3241798-4), Alexa 488 anti-rat antibody (Invitrogen, CatNo:A11006, LotNo: 52955A), Alexa 594 anti-Goat antibody (Invitrogen, CatNo: A32758, LotNo: UH288489), anti-alpha tubulin (Abcam, CatNo: ab125267, LotNo: GR23235-3), horseradish peroxidase (HRP)-conjugated anti-mouse antibody (Cell Signaling Technology, CatNo: 7076S), HRP-conjugated anti-rabbit antibody (Cell Signaling Technology, CatNo: 7074S).
Validation	The primary antibodies were validated according to the method suggested in each analysis.<br>The CD45 antibody from BD is a transmembrane glycoprotein which is expressed at high levels on the cell surface, and its presence

	distinguishes leukocytes from non-hematopoietic cells. BioLegend's anti mouse Ccr2 (CD192) antibody is primarily expressed on monocytes and macrophages, with some expression on basophils. R&D system's anti human/mouse Trem2 is detected human and muse TREM-2 in direct flow cytometry ELISAs and Western blots. The Arg-1 antibody (clone A1exF5) from Invitrogen recognizes both human and mouse Arginase 1. Abcam's anti TREM2, anti-CD68, and anti-alpha tubulin are used by us since several years. The anti-TREM2 detected on the extracellular EC-domain of TRAM2. The CD68 is a highly expressed by cells in the monocyte lineage, by circulating macrophage, and by tissue macrophages. Tubulin is the major constituent of microtubules. We are usually using the reference protein detection at western blot analysis.<br>All relevant validation is available online at the following website: anti mouse CD45 antibody (https://www.bdbiosciences.com/en-ca/products/reagents/flow-cytometry-reagents/research-reagents/single-color-antibodies-ruo/fitc-rat-anti-mouse-cd45.553080); BV421 anti mouse Ccr2 (https://www.biolegend.com/it-it/products/brilliant-violet-421-anti-mouse-cd192-ccr2-antibody-13001); APC anti human/mouse Trem2 (https://www.rndsystems.com/products/human-mouse-trem2-apc-conjugated-antibody-237920_fab17291a); PE-Cy7 anti human/mouse Arg-1 (https://www.thermofisher.com/antibody/product/Arginase-1-Antibody-clone-A1exF5-Monoclonal/25-3697-82); anti-TREM2 (https://www.abcam.com/trem2-antibody-n-terminal-ab175525.html); anti-CD68 (https://www.abcam.com/cd68-antibody-fa-11-ab53444.html); anti-alpha tubulin (https://www.abcam.com/alpha-tubulin-antibody-ab125267.html).

Animals and other organisms

Policy information about studies involving animals; ARRIVE guidelines recommended for reporting animal research

Laboratory animals	We used male wild-type 7-8 week-old C57BL/6 mice that were purchased from Orient Bio (Gyeonggido, Korea). Mice were housed in a specific pathogen-free facility maintained on a 12 h light-dark cycle at 20-26 °C and 50 ± 10% humidity.
Wild animals	The study did not involve wild animals.
Field-collected samples	The study did not involve samples collected from the field.
Ethics oversight	All animal experiments were conducted in compliance with the approval of the Institutional Animal Care and Use Committee (IACUC) at the Catholic University School of Medicine (CUMC-2018-0035-07).

Note that full information on the approval of the study protocol must also be provided in the manuscript.

Flow Cytometry

Plots

Confirm that:

- [x] The axis labels state the marker and fluorochrome used (e.g. CD4-FITC).
- [x] The axis scales are clearly visible. Include numbers along axes only for bottom left plot of group (a 'group' is an analysis of identical markers).
- [x] All plots are contour plots with outliers or pseudocolor plots.
- [x] A numerical value for number of cells or percentage (with statistics) is provided.

Methodology

Sample preparation	For heart single cell preparation used gentleMACS C-tube (Miltenyi Biotec, USA). Blood sample removed RBC using RBC lysis buffer (QIAGEN, USA). After homogenization, heart digested by collagenase type II solution (Worthington Biochemical Corporation, USA) at a concentration of 500unit/ml in 37'c for 40min. After digestion, cell passed through 40um-cell starainer and added HBSS Buffer for stop enzyme digestion step. Single cell resuspended FACS stain buffer and counting total cell number using EVE cell counter.
Instrument	The cells were sorted FACS analysis using FACSCanto II Flow Cytometry System (BD).
Software	After staining each samples were analysis using Flow-jo software.
Cell population abundance	To specifically collect Trem2hi macrophage subsets, we sorted the immune cells based on CD45 expression, and then the macrophage populations were selected using F4/80 antibody. Ccr2hi macrophages and Trem2hi macrophages, as major subsets in the early and late phases of MI, were then collected and analysis.
Gating strategy	The gates and regions are placed around populations of cells with common characteristics, usually forward scatter (FSC), side scatter (SSC). And we sorted the immune cells based on CD45 expression, and the macrophage population was selected using F4/80 (Positive population). We were analysis of the macrophage subset using the positive cells population (CD45+F4/80+). The gating strategy was illustrated in the Supplementary Fig. 13.

- [x] Tick this box to confirm that a figure exemplifying the gating strategy is provided in the Supplementary Information.
